# Effects of an Eccentric Training Protocol Using Gliding Discs on Balance and Lower Body Strength in Healthy Adults

**DOI:** 10.3390/jcm10245965

**Published:** 2021-12-19

**Authors:** Juan Lopez-Barreiro, Pablo Hernandez-Lucas, Jose Luis Garcia-Soidan, Vicente Romo-Perez

**Affiliations:** Faculty of Education and Sport Sciences, Universidade de Vigo, Campus A Xunqueira, s/n., 36005 Pontevedra, Spain; phernandez@uvigo.es (P.H.-L.); jlsoidan@uvigo.es (J.L.G.-S.); vicente@uvigo.es (V.R.-P.)

**Keywords:** lower-body strength, accelerometry, falls, quality of life, squat jump

## Abstract

Impaired balance and lower body weakness are the main causes of falls, which are considered to be the major cause of fractures and head injuries in the elderly and are recognised as a serious health problem. The aim of this study is to observe the effect of eccentric training, introducing new technologies (gliding discs), on body composition, lower body strength, balance and quality of life. A quasi-experimental study was carried out with 56 healthy participants who were divided into an experimental group (*n* = 31) who underwent the protocol consisting of 12 training sessions and a control group (*n* = 25) who did not undergo the training. Before and after the intervention, all participants underwent a measurement of body composition, the SJ jump, balance with accelerometry and quality of life with the Short Form 12 Health Survey. In the experimental group, statistically significant improvements were found in the variables balance and lower body strength. The application of this training protocol improves lower body strength and the ability to control balance in the adult population.

## 1. Introduction

Balance impairment is considered to be responsible for 10–25% of falls [1]. Another risk factor for falls is lower extremity weakness, and according to Graafmans et al. [2], this weakness is a predictor of future falls [2].

Falls are the major cause of fractures and head injuries in the elderly, especially in women [3]. Moreover, they are recognised as a serious health problem that can cause serious injuries such as fractures or head injuries and even death [4,5].

Known risk factors are age, previous falls, arthritis, cognitive disorders, dependence in daily activities, depression, gait and balance dysfunctions, medication, muscle weakness, and visual and sensory impairments [6,7,8].

Elderly people with a history of falls, due to poor muscle strength, experience fear of a possible subsequent fall that could limit their activities and mobility, which directly affects their quality of life [5].

Older adults are more likely to have a higher level of quality of life in relation to an increase in the frequency and duration of physical activity [9]. On this basis, relationships are also established between physical–functional problems and loss of strength, flexibility, hearing, sight, memory and balance [9]. These factors are the same as fall risk factors. Therefore, a direct relationship can be established between balance, risk of falls and quality of life. On the other hand, in addition to the preventive role of training in young adults, the prevalence of frailty in young and middle-aged adults is similar to that of older adults living in the community [10].

Considering this, and given the rapid increase in the age of the world’s population, falls prevention is of extreme importance. It is essential to establish an effective, simple, safe and low-cost method to reduce the risk of fall occurrence [11]. Knowledge of the relationship between balance, strength and power is important for the identification of individual fall risk factors, because deficits in these neuromuscular components are associated with an increased risk of injury or falls [12]. The WHO further recommends varied and multicomponent physical activity with an emphasis on functional balance and moderate- to higher-intensity muscle strength training, three or more days a week, to improve functional capacity and prevent falls [12].

Several studies have shown that training focused on eccentric muscle contractions is more effective in increasing strength and muscle mass, compared to training focused on concentric muscle contractions [13,14,15,16,17]. The most traditional form of eccentric training is the performance of body weight exercises with a long eccentric contraction [18], that was applied by introducing the gliding discs, which make the exercise more complicated and interesting for those who perform it as they increase the amplitude of movement [19].

Based on this, it seems necessary to design and apply appropriate interventions with programmes that are capable of delaying or reversing the limitations produced by the alteration of balance in order to improve quality of life [20].

It is for these reasons that the protocol described in this paper is presented, with the aim of testing whether it improves balance and lower body strength in a healthy adult population, and with the previous hypothesis that this eccentric training programme using gliding discs will produce positive effects on body composition, lower body strength, balance and quality of life.

## 2. Materials and Methods

### 2.1. Design

A quasi-experimental study was conducted with a control group (CG) in a convenience sample, comparing the scores on the measures of the dependent variables before and after the intervention, both in the experimental group (EG) (they followed the training protocol) and in the CG (they continued with their normal daily activity) to compare the possible effects of the programme.

### 2.2. Participants

A non-probabilistic purposive sampling was carried out at the Faculty of Physical Activity and Sport Sciences of the Universidade de Vigo. Eighty-four volunteers were willing to participate in the study and the following inclusion criteria were applied: (a) age between 18 and 65 years; (b) signing the informed consent form; (c) being able to attend the initial measurements (d) not taking medication or presenting any limiting pathology; exclusion: (a) missing more than two training sessions; (b) not being able to attend the final measurements; (c) varying their lifestyle in relation to physical activity, rest and nutrition.

After applying these inclusion criteria, we found 68 subjects, who were randomly assigned to the EG and CG.

**Figure 1:**
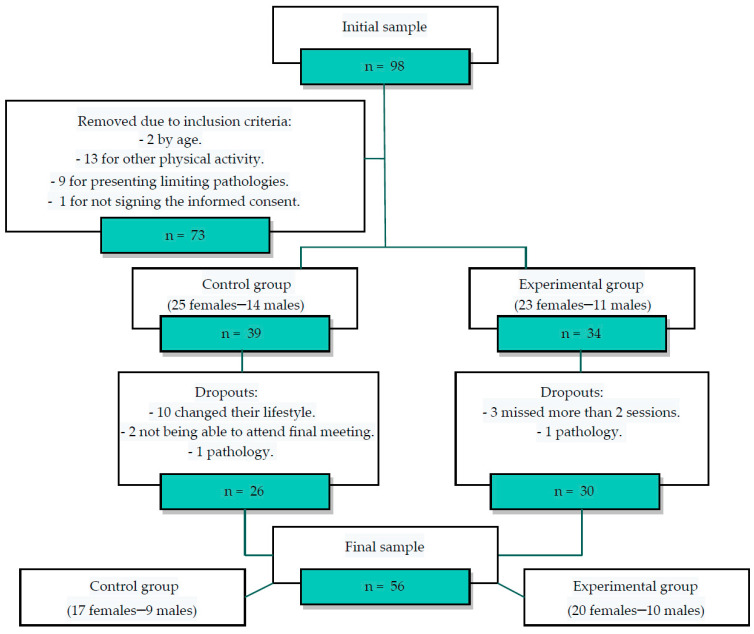
Sample selection flowchart.

There were 12 drop-outs, nine from the CG and three from the EG, with 56 subjects completing the study, leaving the final study sample comprising 31 subjects belonging to the EG and 25 to the CG as shown in the following diagram (Figure 1).

### 2.3. Studied Variables

#### 2.3.1. Anthropometric variables

The anthropometric variables were measured using an OMRON BF-511 scale previously used in research [21,22] and a homologated tallimeter (Seca™ 709, Hamburg, Germany), with the subjects positioned with their heels placed together and their heads in the Frankfort plane. The measurements were taken three times and then averaged.

#### 2.3.2. Lower Body Strength

To measure lower body strength, the ErgoJump Bosco System platform was used to record jump height [23]. This device is a conductive mat (dimensions L-175 × W-70 cm) connected to an electronic timing system. The timer switches on automatically when a subject takes off and switches off as soon as a foot makes contact with the mat again. The test performed was the Squat Jump (SJ). The protocol used for the SJ was as follows: bare feet at hip width, hands on hips, knees bent at 90° and trunk upright, without downward countermovements. Again, subjects performed three jumps from which the average was found.

#### 2.3.3. Quality of Life

The Spanish version of the Short-Form Health Survey SF-12 was used to measure the subjects’ quality of life [24]. This survey was given to the subjects on paper in the measurement sessions before and after the 12 weeks of training.

#### 2.3.4. Balance

To obtain the balance values of each subject, triaxial accelerometers model ActiGraph GT3X+^®^ placed in the lumbar region were used following the measurement protocol described by Leirós-Rodríguez et al. [25].

### 2.4. Intervention

The intervention consisted of an eccentric training programme using gliding discs. It lasted eight weeks, with two sessions per week. A total of 12 training sessions were carried out, lasting approximately 45 min and two sessions to measure the different variables, one at the beginning and other at the end. The training sessions were conducted in small groups organised according to the availability of the participants. Both the measurement sessions and the training sessions were carried out in sports clothes and in an air-conditioned room at a temperature of 22 °C.

#### 2.4.1. Assessment

The measurement sessions were structured as follows, except that the explanation of the study and the reading and signing of the consent form were not repeated in the post-test: (a) explanation of the study to the participants; (b) reading and signing of the informed consent form by the participants; (c) completing the SF-12 questionnaire by the participants; (d) taking anthropometric measurements of the participants; (e) measuring the SJ height; (f) measuring the quality of balance using accelerometry.

#### 2.4.2. Training Sessions

The structure of each training session consisted of a warm-up, a main part and a cool-down.

The warm-up consisted of 6′ walking on a treadmill at 6 km/h with a 1% incline and performing ten repetitions of each of the following joint mobility exercises: left and right ankle internal and external ankle circling, internal and external knee rotations with semi-flexed knees, right and left hip rotations; ten repetitions of the following strength exercises using body weight: half squats, dynamic front splits with left and right leg, dynamic back splits with left and right leg and lateral splits with left and right leg.

The main part consisted of eccentric training using body weight and gliding discs, performing four sets of ten repetitions of the exercises described in Table 1. After eight sessions, the load was increased by increasing the number of repetitions of each exercise by 20%, that is, 12 repetitions.

In the cool down, static stretching was performed for 15” per muscle group, specifically: calf, soleus, quadriceps, hamstrings, psoas iliacus, gluteus, abdominal and lumbar area.

### 2.5. Ethics

The Research Ethics Committee of the Faculty of Education and Sport Sciences of the Universidade de Vigo evaluated and approved the study with registration 04-0721 and all procedures were designed and administered in accordance with the Declaration of Helsinki. An informed consent form was also administered prior to the start of the study.

### 2.6. Statistics Analysis

The normal distribution of the data was verified using the Shapiro–Wilk test and homogeneity of variance with Levene’s test. Both pre-intervention groups were found to show no significant differences in the variables under study with the t-test for independent samples.

An ANOVA 2 × 2 (Group × Momentum) analysis was used to analyse the effects of the intervention for all variables of interest and the effect size was calculated using Cohen’s *d* statistic defined as small: *d* = 0.1; medium: *d* = 0.5; large: *d* = 0.8 [26].

The significance level was set at *p* < 0.05. Analyses were performed with STATA 15.0 for MacOS^®^ software (STATA Corporation, College Station, TX, USA).

## 3. Results

Table 2 details the pre-intervention values. No significant differences were found in any of the variables analysed between the groups at baseline.

Table 3 shows the results of the descriptive analysis of the variables in both groups before and after the intervention (mean and confidence interval). The results obtained in the 2 × 2 ANOVA test and the effect size after the intervention are also shown. In the percentage improvement, it is understood that negative percentages indicate worse results and positive percentages indicate better results between the pre-test and the post-test.

### 3.1. Results of Anthropometric Variables

**Figure 2:**
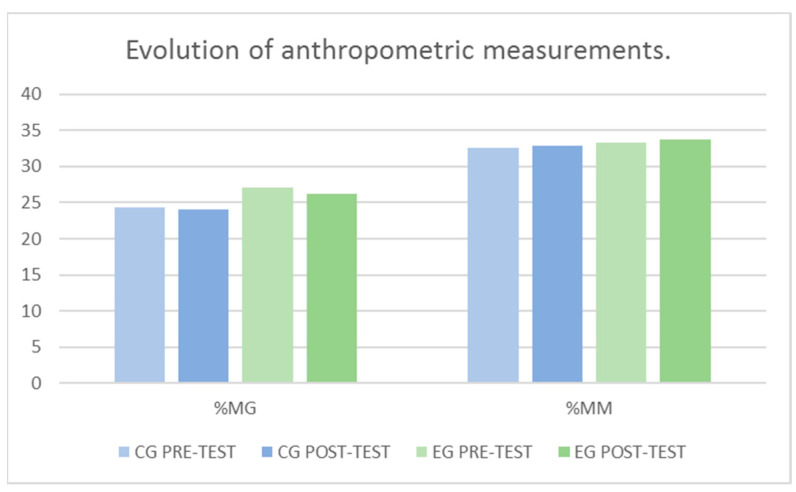
Evolution of the anthropometric measurements between groups and pre-post-test. %MG: body fat percentage; %MM: muscle mass percentage.

The results obtained for %MG shown in Figure 2, indicate that it was not significantly affected after the intervention, with respect to the factor Group *F*_(1–108)_ = 2.64, *p* = 0.107, with respect to the factor Momentum *F*_(1–108)_ = 0.12, *p* = 0.727 and with respect to the interaction (Group × Momentum) *F*_(1–108)_ = 0.05, *p* = 0.818.

The results of the %MM shown in Figure 2 were also not significantly affected after the intervention, with respect to the factor Group *F*_(1–108)_ = 0.61, *p* = 0.438, with respect to the factor Momentum *F*_(1–108)_ = 0.08, *p* = 0.777 and with respect to the interaction (Group × Momentum) *F*_(1–108)_ = 0.01, *p* = 0.933.

### 3.2. Results of the Strength of the Lower Body Musculature (SJ)

**Figure 3:**
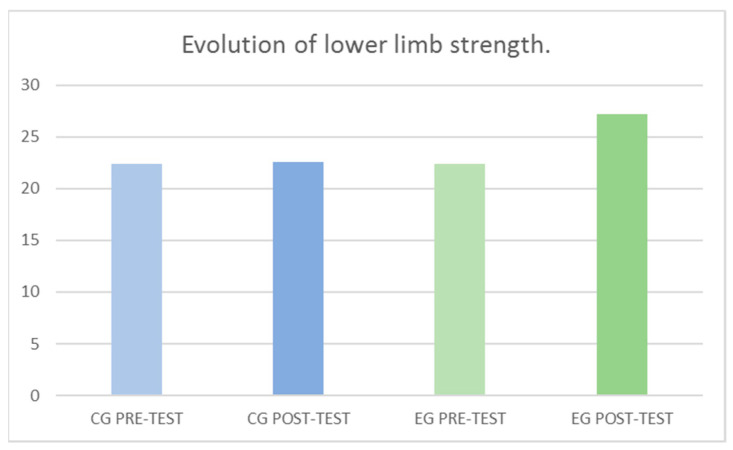
Evolution of lower limb strength between groups and pre-post-test.

In SJ, significant differences were found with respect to the factor Group *F*_(1–108)_ = 4.21, *p* = 0.043, with respect to the factor Momentum *F*_(1–108)_ = 5.04, *p* = 0.027 and with respect to the interaction (Group × Momentum) *F*_(1–108)_ = 4.11, *p* = 0.045. With a medium effect size and percentage improvement of 21.28% in jump height (Table 3). SJ’s results can be observed more graphically in Figure 3.

### 3.3. Results of the Capacity to Control Balance (EQUI)

**Figure 4:**
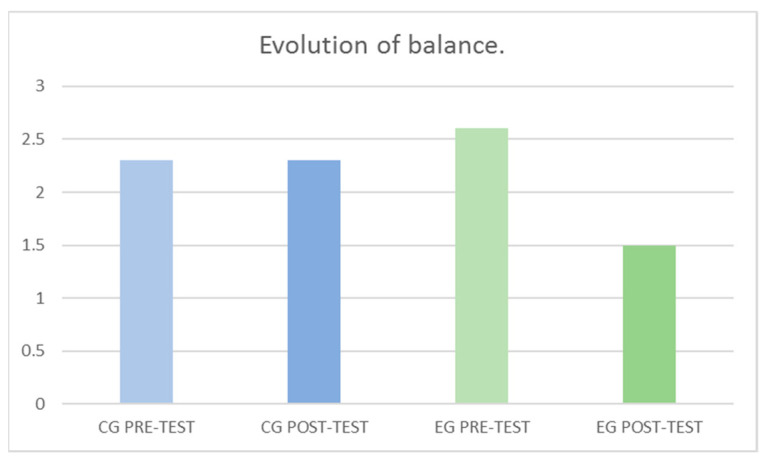
Evolution of the capacity to control balance between groups and pre-post-test.

In EQUI, as detailed in Table 3, significant differences were found for the factor Group *F*_(1–108)_ = 1.48, *p* = 0.222, for the factor Momentum *F*_(1–108)_ = 6.86, *p* = 0.010 and for the interaction (Group × Momentum) *F*_(1–108)_ = 7.43, *p* = 0.008, with a large effect size and a percentage of improvement of 59%. These results can be observed more graphically in Figure 4.

### 3.4. Results of the Quality of Life (QoL)

**Figure 5:**
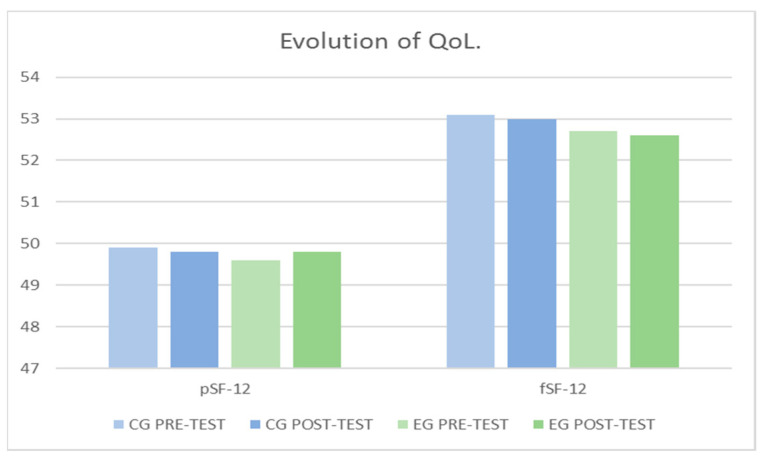
Evolution of the two dimensions of QoL between groups and pre-post-test.

The results obtained for pSF-12, shown in Figure 5, indicate that it was not significantly affected after the intervention, with respect to the factor Group *F*_(1–108)_ = 0.59, *p* = 0.654, with respect to the factor Momentum *F*_(1–108)_ = 0.59, *p* = 0.654 and with respect to the interaction (Group × Momentum) *F*_(1–108)_ = 0.03, *p* = 0.862 (Table 3).

The results obtained for fSF-12, shown in Figure 5, indicate that it was not significantly affected after the intervention, with respect to the factor Group *F*_(1–108)_ = 0.64, *p* = 0.424, with respect to the factor Momentum *F*_(1–108)_ = 0.02, *p* = 0.899 and with respect to the interaction (Group × Momentum) *F*_(1–108)_ = 0.00, *p* = 0.958 (Table 3).

## 4. Discussion

The aim of this study was to test if the proposed eccentric training protocol improves body composition, lower body strength, balance and quality of life in a healthy adult population. The results suggest a positive effect on the parameters analysed, highlighting the differences obtained in the jumping and balance tests obtained in the EG.

For the other variables, %MG, %MM and SF-12 (fSF-12 and pSF-12), no significant differences were obtained for the sample analysed.

In reference to anthropometric variables such as %GM and %MM, no significant improvements were achieved in accordance with the findings shown in other studies [15,22] which stated that improvements in weight and BMI were achieved with moderate intensity training, although in their case with a study of a much longer duration and conducted with subjects suffering from obesity with an average BMI of 38 points, which by WHO classification is considered class II obesity, and a stair descent protocol with obese women with a BMI of 26.2 and a %MG of 36.4%, respectively; our subjects had an average BMI of 24.9, being at the upper limit of what this organisation considers normal values and a similar %MG with an average score of 25.5%.

Motalebi et al. [11] and Gordon et al. [27] report in their work that eccentric strength training increases strength levels, in this case of the lower body musculature. As in our case, using the SJ, we measured jump height, which is an indirect measure of the strength of the lower body musculature. In the study of Gordon et al. [27], they proposed an eccentric strength training protocol compared to a traditional leg press of two sessions per week for four weeks, and they said that these levels improved. In their case, they observed improvements both at two and four weeks, that is, with four and eight sessions; in our case, the results were evaluated after twelve training sessions, obtaining an improvement of 21.28%.

Regarding the results obtained on balance, they support the hypothesis that an eccentric training programme using gliding discs would improve balance, as they showed a significant improvement after the intervention. We agree with the results obtained in other studies [11,28], which state that strength work improves the capacity to control balance, as well as being relatively simple and safe. On the other hand, in the training proposed in their research, strength work was achieved using whole-body vibration platforms, Bosus and elastic bands, respectively, instead of the gliding discs used in our protocol, as well as lasting for a shorter time (six weeks) and using a sample of female athletes with chronic ankle instability in the case of Chang et al. [28] and lasting longer and using a smaller sample size in the EG in the case of Motalebi et al. [11].

These findings are also consistent with those shown in the work of Olson et al. [1], who found significant improvements in the capacity to control balance in older people with a home training programme.

Regarding the results of the quality of life obtained by the SF-12 survey, we started from values similar to the average of the Spanish population for this age group, both in the physical and psychosocial dimensions [29]. These values were not significantly improved after the application of the training protocol.

These results are consistent with the findings of an extensive systematic literature review and meta-analysis, which analysed 37 articles and concluded that there is a high correlation between strength and balance [12]. This is similar to our work, since both the parameters of lower body strength measured by means of the SJ jump and the ability to control balance were significantly improved after the intervention (21.28 and 59%, respectively), so we can affirm that the eccentric training protocol using gliding discs proposed in this study serves to increase the strength of the musculature of the lower body and to improve the ability to control balance in healthy middle-aged subjects.

A limitation of our study is the diversity in the age of the participants, as well as not stratifying the results by age and gender due to the small number of participants. Another limitation is the restrained number of participants. In terms of future research, it would be interesting to use a much larger sample or to conduct research by age range and gender. It would also be interesting to do a long-term follow-up to see if the risk of falls was reduced. However, as far as we know, this is the first study in which gliding discs have been used for this purpose, and it is important to integrate new technologies in training for fall prevention.

## 5. Conclusions

The implementation of this eccentric training programme using gliding discs, of 12 sessions of 45′ duration, produced an increase in lower body strength and improved the ability to control balance, which may reduce the probability of falls in a healthy adult population. On the other hand, no improvements in body composition or quality of life were obtained. It should be noted that this type of training can be performed both in gyms and at home without the use of a large amount of equipment, with the consequent socioeconomic benefit in terms of time and money.

## Figures and Tables

**Table 1:** Main part of the training.

Exercise	Starting/Ending Position	Middle Position
Back split	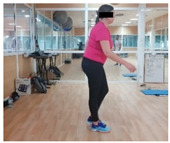	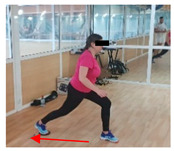
Lateral split	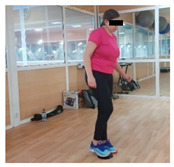	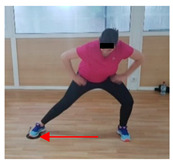
Front split	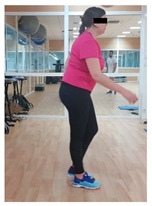	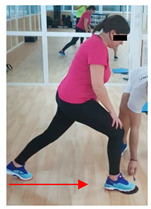
Hamstring curl	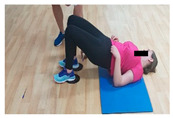	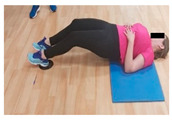

**Table 2:** Baseline of the studied variables.

Variable	ALL (*n* = 56)		CG (*n* = 25)		EG (*n* = 31)		*p*-Value
	x ± SD	Median	x ± SD	P50	x ± SD	Median	
Age (Years)	30.9 ± 10.5	27.5	30.6 ± 8.9	28	31.2 ± 11.8	27	0.827
Weight (Kg)	72.1 ± 12.7	71	69.8 ± 11.5	69	74 ± 13.5	71.1	0.313
Height (cm)	1.7 ± 0.1	1.7	1.7 ± 0.1	1.68	1.7 ±0.1	1.7	0.219
BMI (Kg/m^2^)	24.9 ± 2.7	24.7	24.5 ± 2.4	24.62	25.2 ± 3.1	24.5	0.359
%MG	25.6 ± 7.9	23.8	24.3 ± 6.9	22.6	27.1 ± 9.1	27.7	0.207
%MM	33.1 ± 5.6	32.4	32.6 ± 5	31.5	33.3 ± 6.4	33.1	0.633
SJ	23.8 ± 6.2	23.8	22.4 ± 5.6	21.3	22.4 ± 6.7	21.9	0.987
EQUI	2.2 ± 1.1	2	2.3 ± 1.2	1.99	2.6 ± 1.2	2.4	0.339
mSF-12	50.2 ± 1.9	51	49.9 ± 2.1	51	49.6 ± 2.3	51	0.594
fSF-12	52.8 ± 2.8	53	52.1 ± 2.4	53	52.7 ± 3.3	53	0.611

SD: standard deviation; BMI: body mass index; %MG: body fat percentage; %MM: muscle mass percentage; SJ: squat jump; EQUI: capacity to control balance; fSF-12: physical dimension of the survey SF-12; mSF-12: mental dimension of the survey SF-12.

**Table 3:** Inferential statistics of the 2 × 2 ANOVA test and effect sizes.

Variable	Group	Pre-Test Mean 95% CI		Post-Test Mean 95% CI		Cohen’s d	Group M Group × M *p*-Value		
%MG	CG	24.3	(21.4–27.2)	24.1	(21.2–26.9)	−0.10	0.107	0.727	0.818
	EG	27.1	(23.8–30.4)	26.2	(23.2–29.3)				
%MM	CG	32.6	(30.5–34.6)	32.8	(30.7–34.9)	0.06	0.438	0.777	0.933
	EG	33.3	(30.9–35.6)	33.7	(31.5–35.9)				
SJ	CG	22.4	(20.1–24.8)	22.6	(20.6–24.7)	0.76	0.043	0.027	0.045
	EG	22.4	(19.9–24.9)	27.2	(25.1–29.3)				
EQUI	CG	2.3	(1.8–2.8)	2.3	(1.9–2.8)	−1.11	0.226	0.010	0.008
	EG	2.6	(2.2–3)	1.5	(1.3–1.8)				
pSF-12	CG	49.9	(48.6–51.1)	49.8	(48.4–50.9)	−0.01	0.654	0.754	0.862
	EG	49.6	(48.4–50.9)	49.8	(48.5–50.7)	0.02			
fSF-12	CG	53.1	(52.1–54.1)	53	(51.9–54.1)	−0.03	0.424	0.899	0.958
	EG	52.7	(51.5–53.9)	52.6	(51.6–53.6)				

SD: standard deviation; BMI: body mass index; %MG: body fat percentage; %MM: muscle mass percentage; SJ: squat jump; EQUI: capacity to control balance; fSF-12: physical dimension of the survey SF-12; mSF-12: mental dimension of the survey SF-12.

## Data Availability

The datasets generated during and analysed during the current study are available from the aim author or the corresponding author on reasonable request.
